# Adverse event management in the TOURMALINE-MM3 study of post-transplant ixazomib maintenance in multiple myeloma

**DOI:** 10.1007/s00277-020-04149-5

**Published:** 2020-07-01

**Authors:** Martin Kaiser, Meral Beksaç, Nina Gulbrandsen, Fredrik Schjesvold, Roman Hájek, Philippe Moreau, Felipe de Arriba de la Fuente, María-Victoria Mateos, Sharon West, Andrew Spencer, S. Vincent Rajkumar, Kaveri Suryanarayan, Michael Czorniak, Cong Li, Zhaoyang Teng, Richard Labotka, Meletios A. Dimopoulos

**Affiliations:** 1 Department of Haematology, The Royal Marsden Hospital, London, UK; 2 Division of Molecular Pathology, The Institute of Cancer Research (ICR) and The Royal Marsden Hospital, 123 Old Brompton Road, London, SW7 3RP UK; 3 Department of Hematology, Ankara University, Ankara, Turkey; 4 Oslo Myeloma Center, Oslo University Hospital, and KG Jebsen Center for B Cell Malignancies, University of Oslo, Oslo, Norway; 5 Department of Hematooncology, University Hospital Ostrava, Ostrava, Czech Republic; 6 Department of Hematology, University Hospital Hôtel-Dieu, Nantes, France; 7 Servicio de Hematología y Oncología Médica, Hospital Universitario Morales Meseguer y Centro Regional de Hemodonación, IMIB-Arrixaca, Universidad de Murcia, Murcia, Spain; 8 Department of Hematology, University Hospital of Salamanca, CIC, IBM CC, Salamanca, Spain; 9 Malignant Haematology and Stem Cell Transplantation Service, Alfred Health-Monash University, Melbourne, Australia; 10 Division of Hematology, Department of Internal Medicine, Mayo Clinic, Rochester, MN USA; 11 Millennium Pharmaceuticals, Inc., a wholly owned subsidiary of Takeda Pharmaceutical Company Limited, Cambridge, MA USA; 12 Hematology and Medical Oncology, Department of Clinical Therapeutics, School of Medicine, National and Kapodistrian University of Athens, Athens, Greece

**Keywords:** Adverse events, Ixazomib, Maintenance therapy, Multiple myeloma, Safety

## Abstract

The phase 3, double-blind, placebo-controlled TOURMALINE-MM3 study (NCT02181413) demonstrated improved progression-free survival with ixazomib maintenance versus placebo post autologous stem cell transplant (ASCT) in multiple myeloma patients. We report additional safety data from TOURMALINE-MM3 to inform adverse event (AE) management recommendations. Patients were randomized 3:2 to receive ixazomib (*n =* 395) or placebo (*n =* 261) on days 1, 8, and 15 of 28-day cycles for ~ 2 years or until progressive disease/toxicity. The initial 3-mg ixazomib dose was escalated to 4 mg in cycle 5, if tolerated in cycles 1–4. Safety was a secondary endpoint assessed in all treated patients; AEs were graded using Common Terminology Criteria for AEs v4.03. The rate of grade ≥ 3 AEs was higher in the ixazomib arm (19%) than in the placebo arm (5%), but the rate of discontinuation due to AEs was similar (7% vs. 5%). For AEs of clinical interest, rates were higher with ixazomib versus placebo: nausea 39% versus 15%, vomiting 27% versus 11%, diarrhea 35% versus 24%, thrombocytopenia 13% versus 3%, and peripheral neuropathy 19% versus 15%. However, the majority of events were low-grade, manageable with supportive therapy or dose reduction, and reversible, and did not result in discontinuation. There was no evidence of cumulative, long-term, or late-onset toxicity with ixazomib maintenance. Ixazomib is an efficacious and tolerable option for post-ASCT maintenance. AEs associated with ixazomib maintenance can be managed in the context of routine post-ASCT supportive care due to the limited additional toxicity. ClinicalTrials.gov NCT02181413

## Introduction

In patients with newly diagnosed multiple myeloma (MM), maintenance therapy is increasingly used following autologous stem cell transplant (ASCT) in order to delay relapse arising from residual disease [1, 2]. The goals of maintenance treatment are to prolong or deepen response and to extend progression-free survival (PFS) and overall survival (OS) without chronic toxicity, unmanageable adverse events (AEs), or deterioration of quality of life.

Lenalidomide is approved as maintenance therapy following ASCT and is the current standard of care in this setting [1, 3]. Approval was based on a meta-analysis of results from three randomized trials that demonstrated improved PFS with lenalidomide maintenance versus placebo or no treatment [4–8]. The impact of lenalidomide maintenance on OS varied across the individual trials; however, the meta-analysis demonstrated an overall improvement in OS [8]. The most frequently reported AEs with lenalidomide maintenance across all trials were hematologic [4–7], and guidelines for blood count monitoring and dose modification for grade 3/4 neutropenia and thrombocytopenia are provided in the prescribing information [3]. The risk of development of secondary primary malignancies varied across studies, but an increased risk was demonstrated with lenalidomide maintenance versus placebo in the meta-analysis [4, 5, 8, 9].

Ixazomib, the first oral proteasome inhibitor [10], is approved in combination with lenalidomide-dexamethasone (Rd) for the treatment of patients with multiple myeloma who have received at least one prior therapy [11]. Approval of ixazomib was based on the results of the phase 3 TOURMALINE-MM1 study, which showed a significant PFS benefit for ixazomib-Rd versus placebo-Rd in patients with relapsed or refractory multiple myeloma, with limited additional toxicity [12]. Common AEs reported more frequently with ixazomib-Rd versus placebo-Rd included thrombocytopenia, gastrointestinal toxicities, rash, and peripheral neuropathy (PN); toxicities were generally manageable with supportive care and dose delays/reductions as required [13].

The efficacy and safety of ixazomib as maintenance therapy following ASCT for patients with multiple myeloma have been investigated in the phase 3, multicenter, randomized, double-blind, placebo-controlled TOURMALINE-MM3 trial (NCT02181413) [14]. In the primary analysis from this trial (median follow-up, 31 months), ixazomib maintenance significantly improved PFS compared with placebo, resulting in a 28% reduction in the risk of progression or death (median PFS, 26.5 vs. 21.3 months; hazard ratio, 0.72 [95% confidence interval, 0.58, 0.89]; *P =* 0.0023) [14]. The placebo-controlled study design of TOURMALINE-MM3 allowed for careful assessment of AEs in the post-transplant maintenance setting, and ixazomib maintenance was demonstrated to be well-tolerated, with a low rate of treatment discontinuations due to AEs. Here, we report additional safety data from TOURMALINE-MM3 to inform AE management recommendations for clinical practice, focusing on AEs known to impact patient quality of life and treatment adherence.

## Patients and methods

The design of the TOURMALINE-MM3 study has been described previously [14]. Briefly, eligible patients were adults with a confirmed diagnosis of symptomatic MM (by the International Myeloma Working Group criteria) who had achieved at least a partial response after receiving standard-of-care induction therapy (including a proteasome inhibitor and/or an immunomodulatory drug) followed by high-dose melphalan conditioning and single ASCT. Following transplant, patients were randomized in a 3:2 ratio to receive either oral ixazomib 3 mg (*n* = 395) or matching placebo (*n* = 261) on days 1, 8, and 15 in 28-day cycles. Stratification factors were induction regimen, pre-induction disease stage, and post-transplant response. The dose of ixazomib was increased to 4 mg starting at cycle 5 if ixazomib was tolerated during the previous 4 cycles. Treatment was continued for up to 26 cycles (~ 2 years) or until progressive disease or unacceptable toxicity.

The primary endpoint was PFS. Safety was a secondary endpoint assessed in all patients who received ≥ 1 dose of ixazomib or placebo (safety population; analyzed according to the treatment patients actually received). The type, incidence, and intensity of treatment-emergent AEs were evaluated. AEs were coded using the Medical Dictionary for Regulatory Activities (MedDRA) version 20.0 and were graded according to the National Cancer Institute Common Terminology Criteria for Adverse Events (NCI-CTCAE) version 4.03. All AEs that occurred from administration of the first dose of ixazomib or placebo through 30 days after the last dose were recorded. The intensity of AEs and the relationship to study treatment were determined by the investigator. In the event of AEs considered to be related to the study drug, dose modifications were permitted according to protocol-specified guidelines. The study drug (ixazomib or placebo) could be held or reduced by at least one dose level to 3.0, 2.3, or 1.5 mg, followed by discontinuation for persistent toxicity; no subsequent dose re-escalation was permitted.

Supportive measures consistent with optimal patient care (including myeloid growth factors, erythropoietin, red blood cell and platelet transfusions, prophylaxis for deep vein thrombosis/pulmonary embolism, antibiotics, intravenous immunoglobulin, antiemetics, antidiarrheals, and corticosteroids) could be given throughout the study. Unless there was a clinical contraindication, prophylactic antiviral therapy to prevent reactivation of herpes zoster infection was mandatory following a protocol amendment. Use of concomitant medications (such as prophylaxis or symptomatic treatment), including blood products and supportive therapies, was recorded from the first dose of the study drug through 30 days after the final dose. Concomitant medications were classified according to their preferred term in the World Health Organization Drug Dictionary.

This report focuses on prespecified AEs of clinical importance, which included PN (defined according to the high-level term of “peripheral neuropathies not elsewhere classified”), gastrointestinal toxicities (nausea, vomiting, and diarrhea; defined by their preferred terms), thrombocytopenia (defined by the preferred terms “thrombocytopenia” and “platelet count decreased”), and neutropenia (defined by the preferred terms “neutropenia” and “neutrophil count decreased”), as well as AEs of clinical interest including thromboembolic events (defined by the Standardized MedDRA Queries [SMQs] “embolic and thrombotic events, venous” and “embolic and thrombotic events, arterial”), pneumonia (defined by the high-level term of “lower respiratory tract and lung infections”), and herpes zoster (defined by its preferred term). Resolution/improvement of PN events was assessed; resolution was defined as resolved PN with no subsequent event of the same preferred term occurring on the resolution date, or on the day before and/or the day after; improvement was defined as PN improved by ≥ 1 grade from the maximum grade experienced. Time to resolution of PN was defined as the time from the initial onset date to the resolution date. Time to improvement of PN was defined as the time from the initial onset date of the maximum grade to the first date that the toxicity was below the maximum grade with no subsequent higher grade, or to the resolution date, whichever occurred first.

All safety outcomes are presented using descriptive statistics using SAS version 9.2 (or higher).

TOURMALINE-MM3 was conducted in accordance with the International Conference on Harmonisation Guidelines for Good Clinical Practice and all relevant regulatory requirements. The protocol was approved by an ethics committee/institutional review board at each center. All patients provided written informed consent.

## Results

**Fig. 1:**
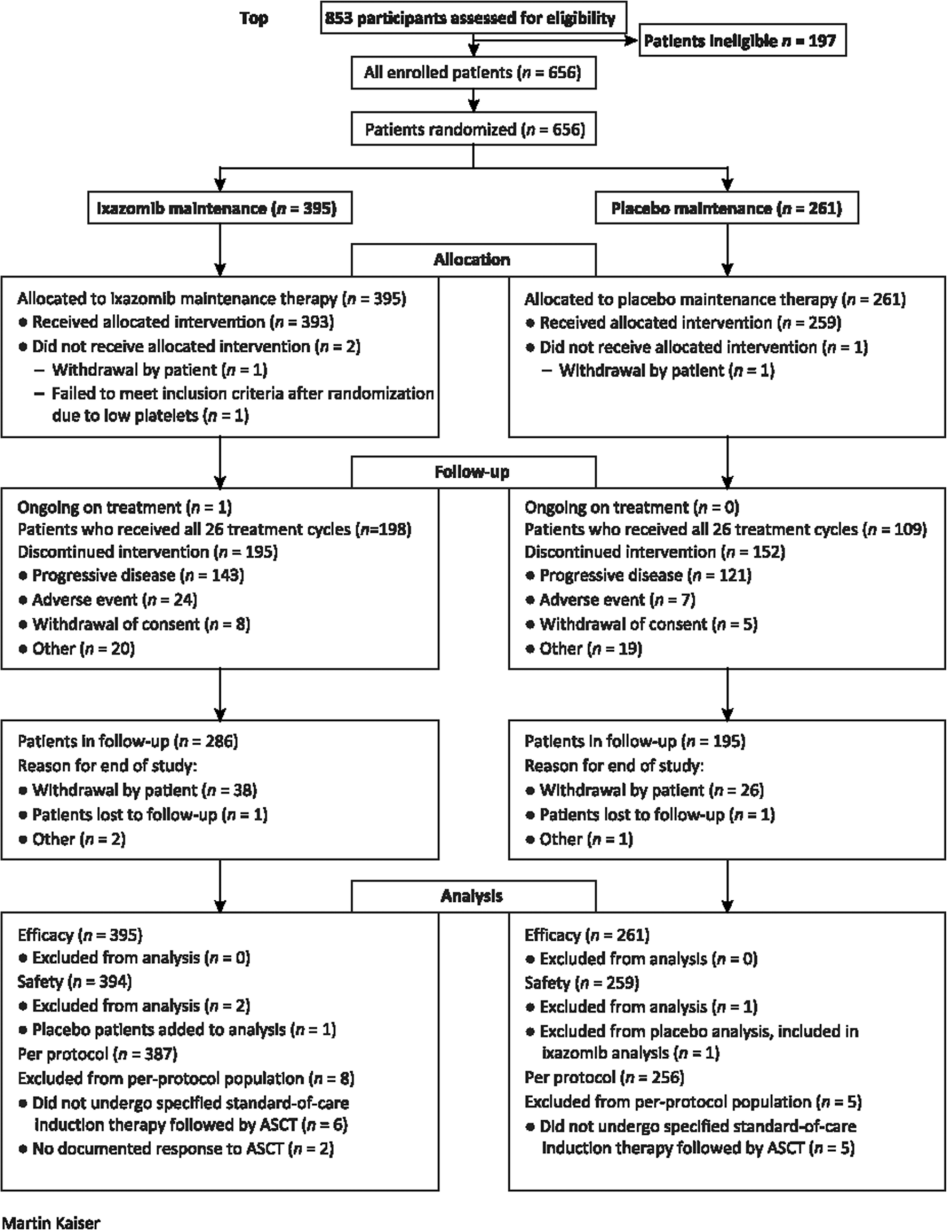
CONSORT diagram. Reproduced with permission from Dimopoulos MA, Gay F, Schjesvold F et al. Oral ixazomib maintenance following autologous stem cell transplantation (TOURMALINE-MM3): a double-blind, randomised, placebo-controlled phase 3 trial. *Lancet* 2019;393:253–264

The safety population comprised 394 patients who received ixazomib maintenance and 259 patients who received placebo (Fig. 1). The median follow-up was 30.9 months in the ixazomib arm and 31.3 months in the placebo arm. The median number of administered treatment cycles was 25 (range, 1–26) for ixazomib and 22 (range, 1–26) for placebo, and the median treatment duration was 713 (range, 8–799) and 629 (range, 13–803) days, respectively; 50% versus 42% of patients completed the full 24 months of treatment. Of the patients who were on ongoing treatment at cycle 5, 317/368 (86%) in the ixazomib arm and 222/242 (92%) in the placebo arm received the 4-mg dose. Median relative dose intensity among all patients was 95% and 99% with ixazomib and placebo, respectively.

As reported previously [14], AEs of any grade were experienced by 97% of patients in the ixazomib arm and 93% of patients in the placebo arm. Rates of drug-related AEs (78% vs. 58%), grade ≥ 3 AEs (42% vs. 26%), drug-related grade ≥ 3 AEs (19% vs. 5%), serious AEs (SAEs; 27% vs. 20%), and AEs resulting in dose reduction (19% vs. 5%) were numerically higher with ixazomib versus placebo. However, the incidence of AEs resulting in treatment discontinuation was similar in both arms (7% vs. 5%). One patient (< 1%) in the ixazomib arm died during the study; there were no on-study deaths in the placebo arm. The most common AEs and AEs of clinical importance are shown in Table 1.

**Table 1:** Common AEs and AEs of clinical importance

AE, *n* (%)	Ixazomib (*n =* 394)				Placebo (*n =* 259)			
	Any grade	Grade ≥ 3	SAE	D/C	Any grade	Grade ≥ 3	SAE	D/C
Hematologic AEs								
Thrombocytopenia^a^	53 (13)	19 (5)	1 (< 1)	2 (< 1)	8 (3)	2 (< 1)	1 (< 1)	0
Neutropenia^a^	36 (9)	20 (5)	0	0	20 (8)	9 (3)	0	0
Anemia	29 (7)	4 (1)	0	0	10 (4)	2 (< 1)	1 (< 1)	0
Non-hematologic AEs								
Infections and infestations								
Upper RTI	101 (26)	2 (< 1)	2 (< 1)	0	54 (21)	1 (< 1)	0	0
Viral upper RTI	94 (24)	0	0	0	69 (27)	0	1 (< 1)	0
Gastrointestinal AEs								
Nausea	154 (39)	1 (< 1)	0	0	40 (15)	0	0	0
Diarrhea	137 (35)	10 (3)	4 (1)	1 (< 1)	61 (24)	2 (< 1)	0	0
Vomiting	106 (27)	6 (2)	1 (< 1)	0	28 (11)	0	0	0
Rash^a^	120 (30)	7 (2)	2 (< 1)	2 (< 1)	57 (22)	0	0	0
AEs of clinical interest								
PN^a^	73 (19)	1 (< 1)	0	2 (< 1)	39 (15)	0	0	2 (< 1)
Thromboembolic AEs								
Venous	2 (< 1)	0	1 (< 1)	1 (< 1)	0	0	0	0
Arterial	1 (< 1)	0	0	0	3 (1)	1 (< 1)	2 (< 1)	0
Pneumonia^b^	40 (10)	25 (6)^b^	24 (6)	2 (< 1)	21 (8)	11 (4)	10 (4)	0
Herpes zoster	39 (10)	3 (< 1)	4 (1)	0	14 (5)	3 (1)	2 (< 1)	0

*AE* adverse event, *D/C* discontinuation due to AE, *MedDRA* Medical Dictionary for Regulatory Activities, *PN* peripheral neuropathy, *RTI* respiratory tract infection, *SAE* serious adverse event, *SMQ* standardized MedDRA query, *SOC* system organ class

^a^Data were based on a SMQ that incorporated pooled preferred terms or multiple preferred terms. Thrombocytopenia included the preferred terms of thrombocytopenia and decreased platelet count. Neutropenia included the preferred terms of neutropenia and decreased neutrophil count. PN represents the high-level term of “peripheral neuropathies not elsewhere classified,” excluding neuritis; preferred terms included “neuropathy peripheral,” peripheral sensory neuropathy, peripheral sensorimotor neuropathy, and peripheral motor neuropathy. Rash included the preferred terms of pruritus, rash maculo-papular, rash macular, rash papular, rash erythematous, rash pruritic, drug eruption, pruritus generalized, rash, urticaria, dermatitis allergic, rash generalized, dermatitis acneiform, erythema multiforme, rash pustular, and rash vesicular

^b^One patient in the ixazomib group had a grade 5 adverse event of pneumonia

### Thrombocytopenia

**Fig. 2:**
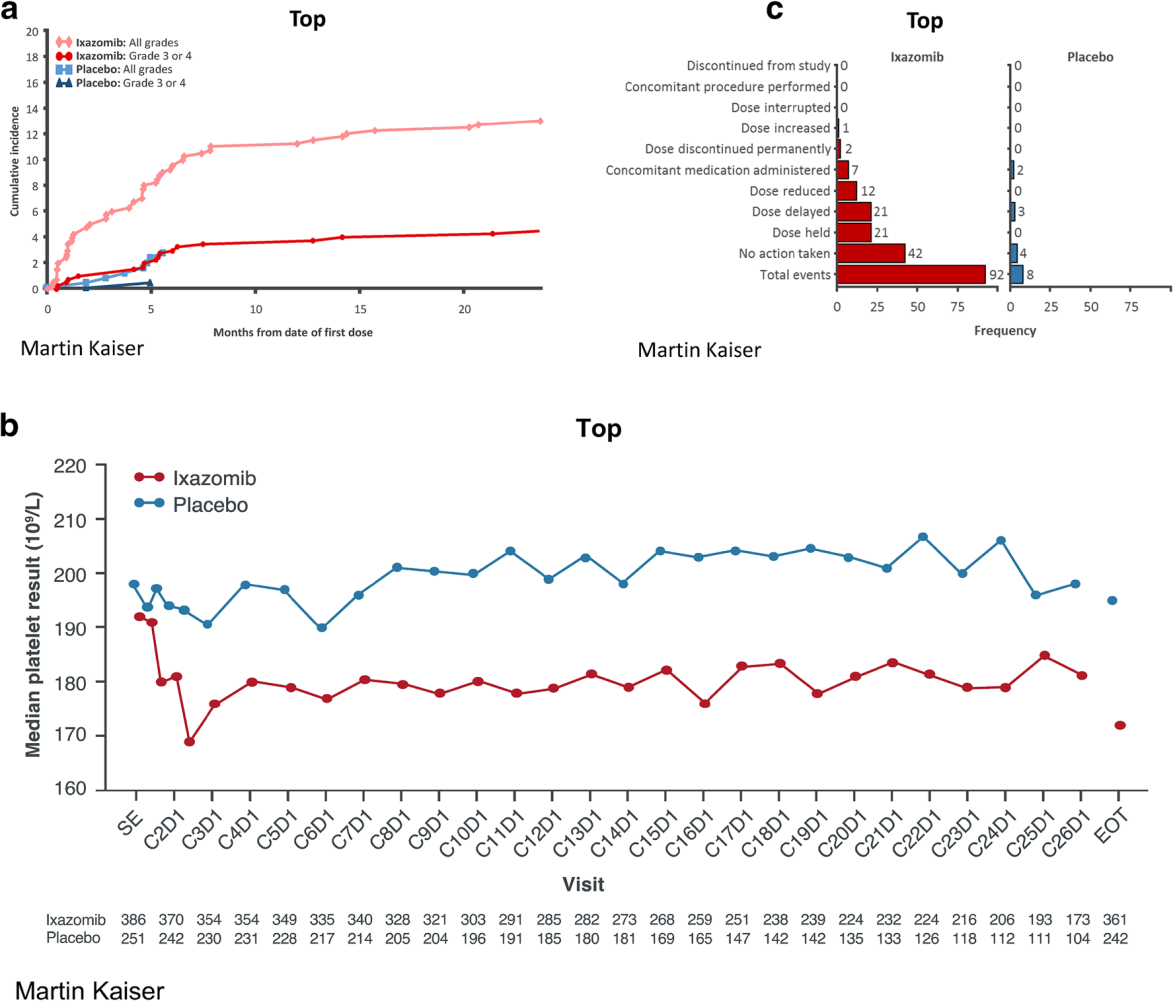
Cumulative incidence of new-onset thrombocytopenia (**a**) and median platelet counts (**b**) over time in the ixazomib and placebo groups, and actions taken for events of thrombocytopenia (**c**). Note: More than one action could be taken for a single event of thrombocytopenia

Thrombocytopenia was more common with ixazomib (13%) versus placebo (3%), and the majority of these patients experienced low-grade thrombocytopenia (Table 1). One patient in each treatment arm had an SAE (both were grade 4 events). In the ixazomib arm, 11 patients (3%) had a dose reduction for thrombocytopenia, and 2 patients (1 each) discontinued due to grade 2 and grade 3 thrombocytopenia. In the placebo arm, no patients had dose reductions or discontinued due to thrombocytopenia. In the ixazomib arm, new-onset thrombocytopenia was most common in the first 6 months of treatment (Fig. 2a). Platelet counts were consistently lower with ixazomib versus placebo, but median counts remained within the normal range (Fig. 2b). Platelet counts followed a cyclical pattern, with a nadir at day 15 of each cycle and recovery by the start of the next cycle; there was no evidence of cumulative thrombocytopenia. Of 92 individual events of thrombocytopenia in patients receiving ixazomib, 42 did not require intervention. When intervention was required, common actions included dose delays or dose holds (Fig. 2c). Seven (2%) and 3 (1%) patients in the ixazomib and placebo arms received platelet transfusions (1 patient receiving placebo had a platelet transfusion but did not have thrombocytopenia). Treatment-emergent AEs within the “hemorrhage” SMQ were reported in 33 (8%) and 15 (6%) patients in the ixazomib and placebo arms, respectively. In the ixazomib arm, 2 patients (< 1%) had grade ≥ 3 events, 3 (< 1%) had SAEs, and 1 (< 1%) discontinued.

### Neutropenia

The incidence of neutropenia was similar in both treatment arms (9% ixazomib vs. 8% placebo), and the incidence of febrile neutropenia was low and similar in both arms (< 1% vs. 0%). Grade ≥ 3 neutropenia was reported in 5% versus 3% of patients. No patient in either arm experienced an SAE or discontinued ixazomib or placebo due to neutropenia (Table 1), and the incidence of neutropenia resulting in dose reduction was < 1% in both arms. Neutrophil counts were generally within the normal range during treatment, and few patients received growth factors in either arm (4% ixazomib and 2% placebo).

### Gastrointestinal toxicities

The incidences of gastrointestinal AEs were higher with ixazomib (27–39%) than with placebo (11–24%), although rates of grade ≥ 3 gastrointestinal AEs were low in both groups (Table 1). Dose reductions due to gastrointestinal AEs were rare (≤ 2% in both groups) and were due to nausea in 6 (2%) versus 0, vomiting in 8 (2%) versus 0, and diarrhea in 8 (2%) versus 1 (< 1%) patients in the ixazomib versus placebo groups, respectively; only 1 patient (in the ixazomib arm) discontinued treatment due to a gastrointestinal event (grade 1 diarrhea). In the safety population, the rates of use of antiemetics (16% vs. 2%; as prophylaxis or symptomatically after first dose) and intravenous fluids (9% vs. 3%) were higher with ixazomib versus placebo, but the rate of antidiarrheal use was similar (8% vs. 7%). For ixazomib versus placebo, the median duration of antiemetic administration was 295 versus 436 days, and the median duration of antidiarrheal administration was 23.5 versus 9.0 days. Potential complications of gastrointestinal AEs (dehydration, weight loss, or grade ≥ 3 hyponatremia, hypokalemia, or hypomagnesemia) were infrequent (rates were < 1% for each individual event in both arms).

**Fig. 3:**
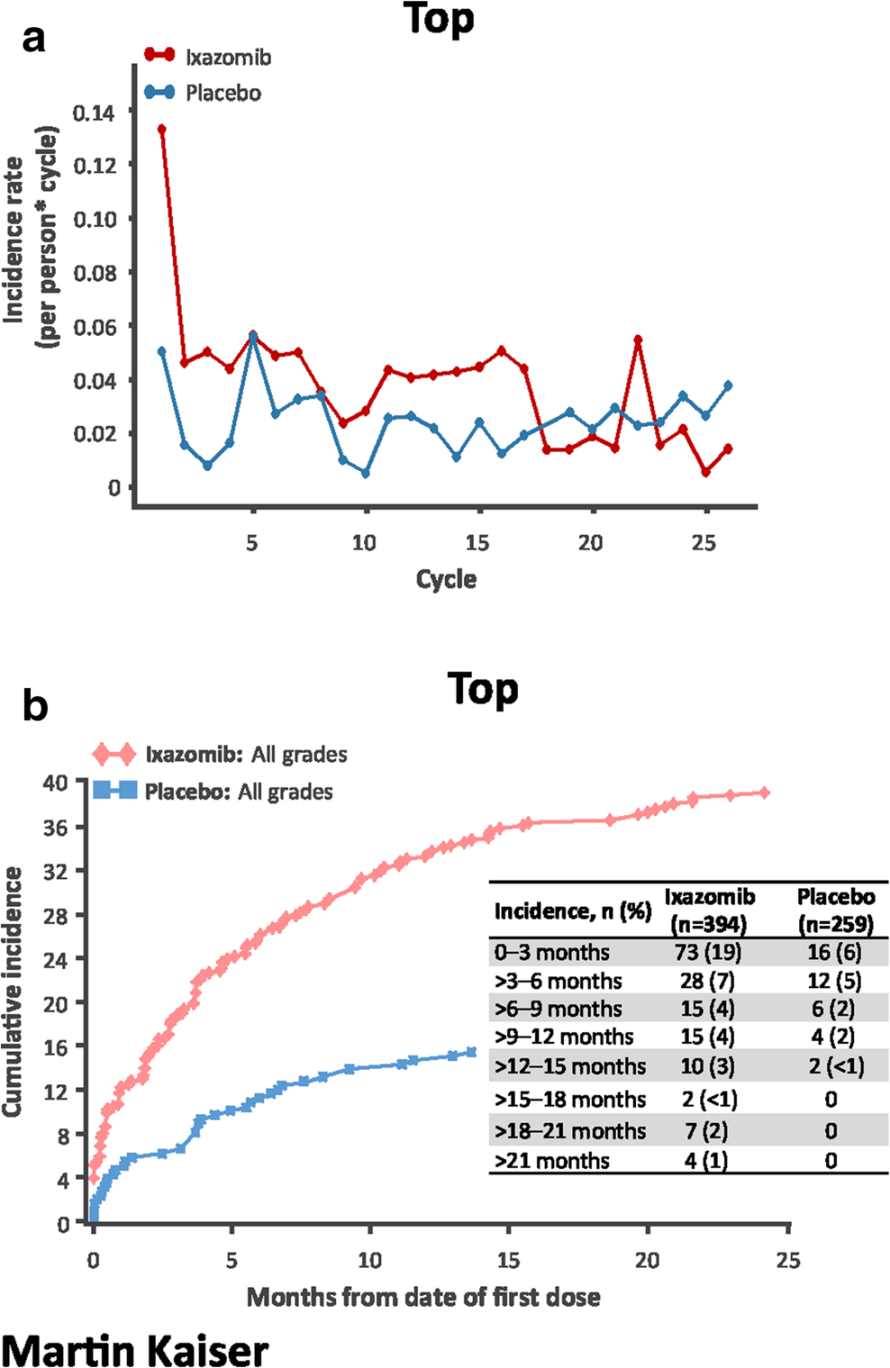
Incidence rate by cycle (**a**) and cumulative incidence (**b**) of new-onset nausea. Only one patient in the ixazomib arm (< 1%) and no patients in the placebo arm had grade ≥ 3 nausea. Incidence rate is the number of events in a cycle divided by the sum of patient cycles at risk in a cycle. A patient with an ongoing AE could not be at risk of getting the same AE until it was resolved

**Fig. 4:**
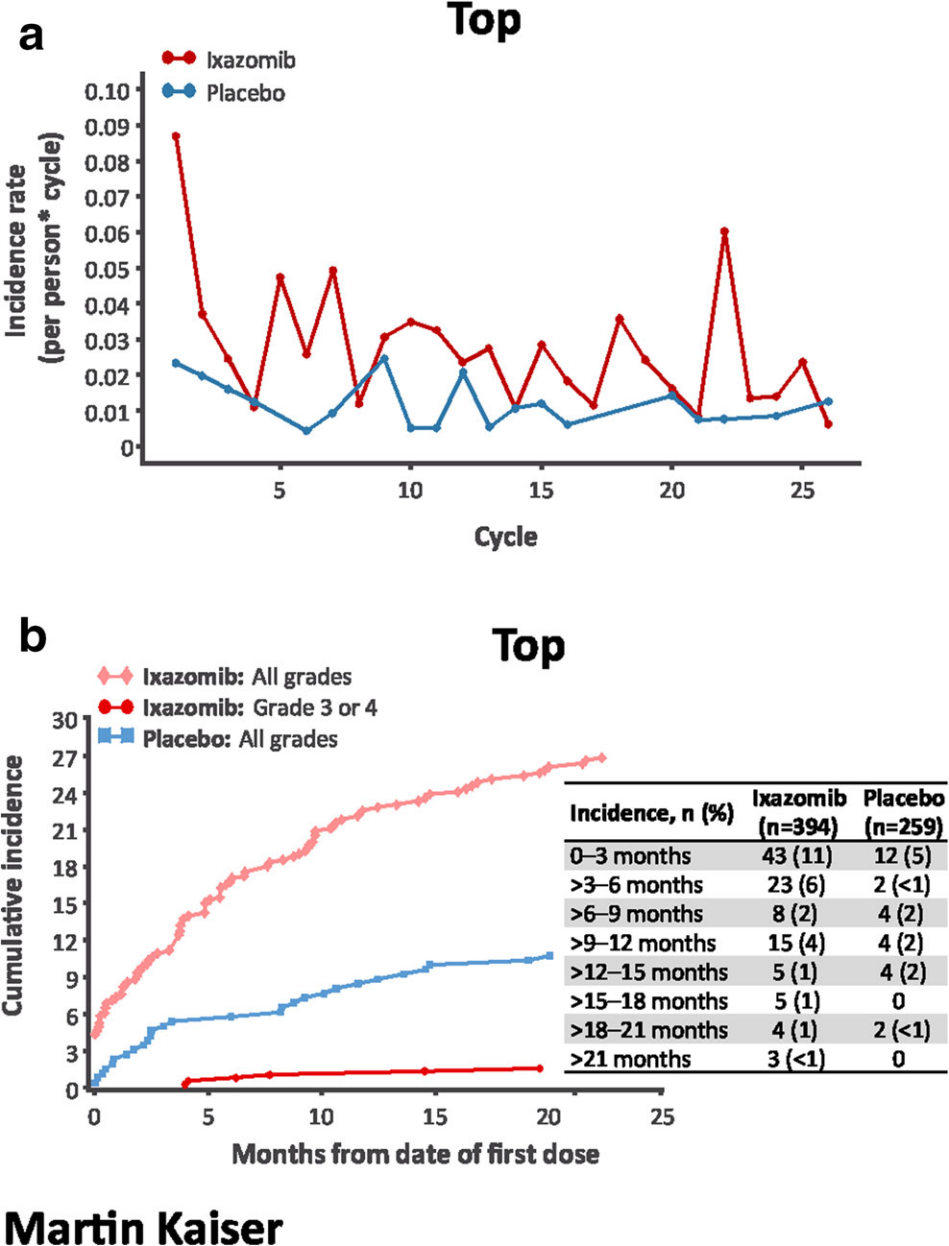
Incidence rate by cycle (**a**) and cumulative incidence (**b**) of new-onset vomiting. No patients in the placebo arm had grade ≥ 3 vomiting. Incidence rate is the number of events in a cycle divided by the sum of patient cycles at risk in a cycle. A patient with an ongoing AE could not be at risk of getting the same AE until it was resolved

**Fig. 5:**
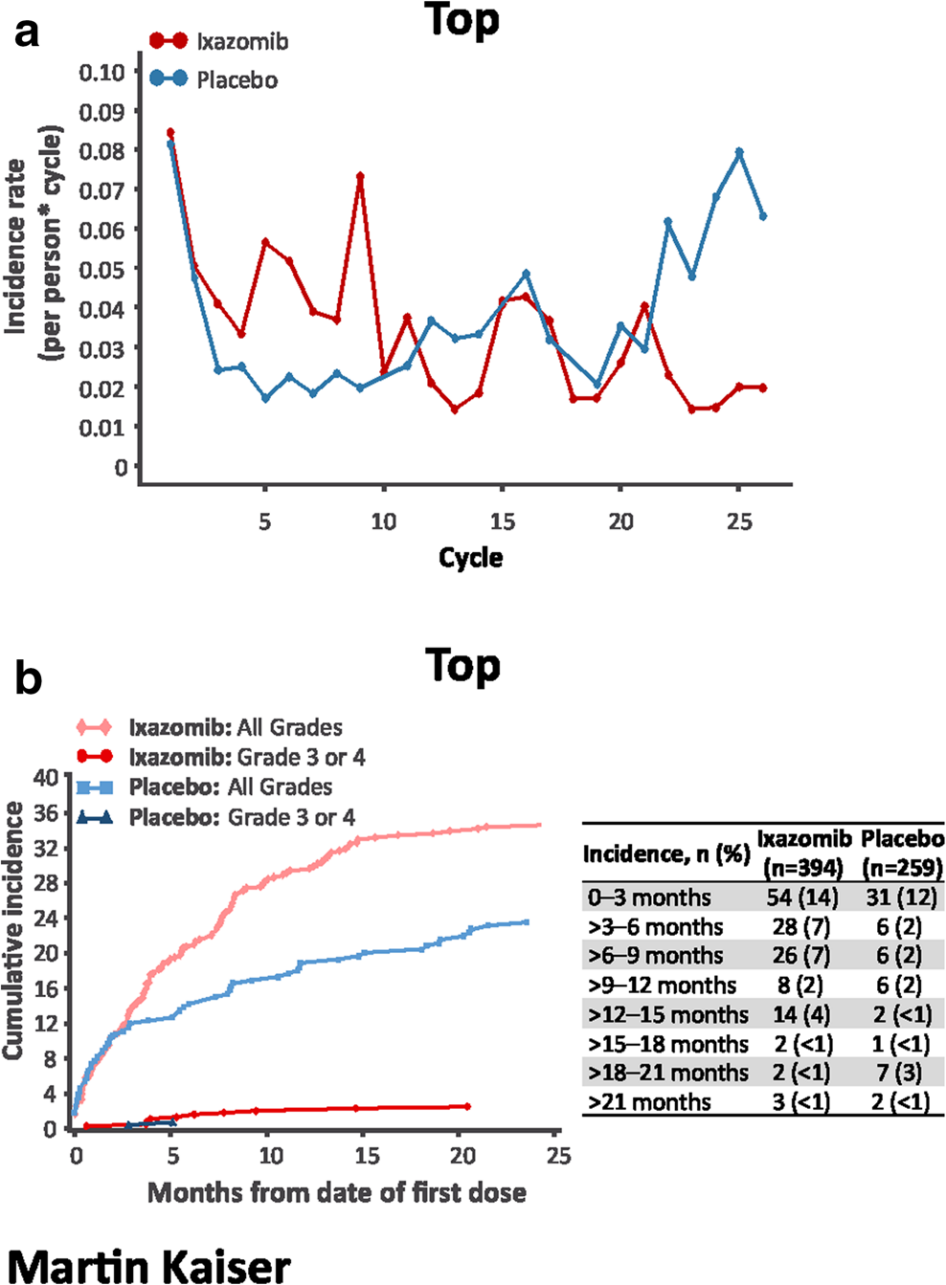
Incidence rate by cycle (**a**) and cumulative incidence (**b**) of new-onset diarrhea. Incidence rate is the number of events in a cycle divided by the sum of patient cycles at risk in a cycle. A patient with an ongoing AE could not be at risk of getting the same AE until it was resolved

The incidence rate of nausea was highest in cycle 1 in the ixazomib arm, and generally higher versus placebo (Fig. 3a). The rate of new-onset nausea was highest during the first 3 months of treatment and then decreased substantially (Fig. 3b). The incidence rate of vomiting was also highest in cycle 1 in the ixazomib arm and generally higher versus placebo (Fig. 4a); new-onset vomiting was most common during the first 3 months and then decreased (Fig. 4b). The incidence rate of diarrhea was similar between groups in cycles 1–2 and then higher with ixazomib versus placebo through cycle 9 (Fig. 5a); the rate of new-onset diarrhea was low and similar between groups thereafter (Fig. 5b).

### Peripheral neuropathy

**Fig. 6:**
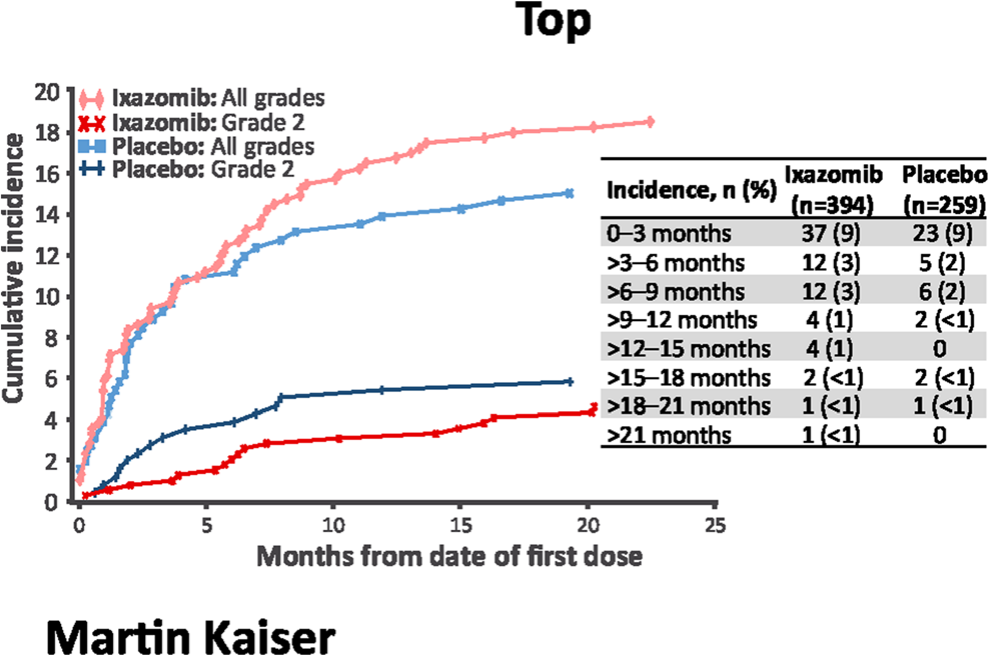
Cumulative incidence of new-onset PN. Only one patient in the ixazomib arm had grade 3 PN (< 1%), and no patients in either arm had grade 4 PN

PN rates were similar in the ixazomib and placebo arms (19% vs. 15%). Only one patient in the ixazomib arm reported grade 3 PN (< 1%), and no patients in either arm had grade 4 PN. In the ixazomib arm, PN appeared more common among patients who were proteasome inhibitor–naïve versus proteasome inhibitor–exposed and among patients with prior thalidomide treatment versus without prior thalidomide treatment (Table 2). In the placebo group, PN appeared less common among proteasome inhibitor–naïve versus proteasome inhibitor–exposed patients, and rates were similar regardless of prior thalidomide treatment (Table 2). The presence of PN at study entry did not affect the rate of treatment-emergent PN in the ixazomib arm but was associated with a higher rate in the placebo arm versus patients without PN at study entry (Table 2). Dose reductions (3% vs. 2%, respectively) and discontinuations (< 1% in both arms) due to PN were uncommon with both ixazomib and placebo (Table 2). Most of the cases of new-onset PN occurred in the first 0–3 (9% in both arms) or 3–6 (3% vs. 2%, respectively) months (Fig. 6). Overall, 73 patients receiving ixazomib reported 94 individual events of PN, and 39 patients receiving placebo reported 43 individual events; the majority of these individual PN events improved (74% vs. 72%) or resolved (70% vs. 65%). The most commonly prescribed concomitant therapies for PN were pregabalin (6/73 patients [8%] in the ixazomib arm, 5/39 patients [13%] in the placebo group) and gabapentin (5/73 [7%] vs. 0/39 [0%]). Median time to PN event improvement was similar in both arms (134 vs. 130 days, respectively). Median time to resolution of PN event was, however, longer with ixazomib than with placebo (225 vs. 159 days, respectively). PN was ongoing in 35 (9%) and 19 (7%) patients in the ixazomib and placebo groups, respectively, at the end of treatment visit; 10 (29%) and 4 (21%) patients subsequently had resolution of their PN events.

**Table 2:** PN: severity, predictive factors, dose modifications, and resolution

	Ixazomib (*n* = 394)	Placebo (*n =* 259)
PN incidence by grade, *n* (%)		
Grade 1	55 (14)	24 (9)
Grade 2	17 (4)	15 (6)
Grade 3	1 (< 1)	0
PN incidence by prior therapy or baseline PN, *n*/*N* (%)		
Prior PI therapy, yes versus no	61/351 (17) versus 12/43 (28)	36/232 (16) versus 3/27 (11)
Prior thalidomide, yes versus no	33/141 (23) versus 40/253 (16)	14/101 (14) versus 25/158 (16)
PN at study entry, yes versus no	8/44 (18) versus 65/350 (19)	12/38 (32) versus 27/221 (12)
Dose reductions due to PN, *n* (%)	10 (3)	5 (2)
Discontinuations due to PN, *n* (%)	2 (< 1)	2 (< 1)
PN—number of individual events, *n*	94	43
Improved, *n* (%)	70 (74)	31 (72)
Median time to improvement (days)	134	130
Resolved, *n* (%)	66 (70)	28 (65)
Median time to resolution (days)	225	159

*PI* proteasome inhibitor, *PN* peripheral neuropathy

### Thromboembolic events

Thromboprophylaxis was not mandated by the protocol but could have been administered per institutional guidelines, and 19% of patients in each arm used an antithrombotic agent. Based on the SMQ for venous embolic and thrombotic events, 2 patients (< 1%) in the ixazomib arm had a thrombotic event. One of these patients had a history of port catheter implantation, and the other patient had a history of pulmonary embolism and was receiving antithrombotic medication. Each of these patients had low-grade events. One of these patients experienced thromboembolic events of jugular vein thrombosis and subclavian vein thrombosis that led to hospitalization and resulted in discontinuation of ixazomib. In the placebo arm, no patient had a venous thromboembolic event. Arterial thromboembolic events, as assessed according to the SMQ for arterial embolic and thrombotic events, were reported infrequently (< 1% ixazomib, 1% placebo).

### Pneumonia

Within the higher-level term of lower respiratory tract and lung infections, the incidence of AEs was higher in the ixazomib arm (23%) versus the placebo arm (18%), with pneumonia (10% vs. 8%) and bronchitis (10% vs. 7%) the most commonly reported preferred terms. The percentages of patients who experienced SAEs (8% ixazomib, 5% placebo) and AEs resulting in discontinuation of ixazomib (< 1%) or placebo (0%) were generally similar in both arms. One patient receiving ixazomib died on study due to pneumonia. The patient was a 61-year-old Asian man with a medical history of diabetes mellitus, hypertension, and hyperlipidemia. The patient had a grade 3 SAE of diarrhea beginning on cycle 6 day 15 and a grade 4 SAE of pneumonia beginning on cycle 6 day 17, both of which led to hospitalization on cycle 6 day 17. During hospitalization, bronchoalveolar lavage was positive for metapneumovirus. The patient developed acute respiratory distress syndrome progressing to multiorgan failure secondary to pneumonia and died due to pneumonia on cycle 6 day 26. The event was considered related to ixazomib. The patient’s last dose of ixazomib prior to the event was taken on cycle 6 day 15.

### Herpes zoster

During the trial, the protocol was amended to mandate prophylaxis for herpes zoster. Overall, 95% of patients in the ixazomib arm and 89% of patients in the placebo arm received direct-acting antivirals. Of the 55 ixazomib-treated patients and the 47 placebo-treated patients who were not receiving appropriate antiviral prophylaxis, 33 (60%) and 12 (26%) reported herpes zoster. For patients who did receive prophylaxis, 6/339 patients (2%) in the ixazomib arm and 2/212 (< 1%) in the placebo arm developed herpes zoster. The most commonly prescribed antiviral medications were aciclovir in 240/394 (61%) and 143/259 (55%) patients, and valaciclovir in 144 (37%) and 84 (32%) patients in the ixazomib and placebo groups, respectively. No patients in either treatment arm discontinued due to herpes zoster.

## Discussion

This safety analysis of TOURMALINE-MM3 demonstrates that maintenance therapy with ixazomib is well-tolerated, with limited additional toxicity compared with placebo. The majority of patients who experienced AEs had events that were low-grade and non-serious, and that did not result in discontinuation. Although the overall incidence of grade ≥ 3 AEs was higher with ixazomib versus placebo, the rate of discontinuation due to AEs was similar between treatment arms. The most frequently reported AEs in the ixazomib arm were nausea, diarrhea, and vomiting, which were generally expected and consistent with the known safety profile of single-agent ixazomib from prior phase 1 and phase 1/2 studies [15–17], although the patient populations and treatment durations differed between these prior studies and TOURMALINE-MM3. Importantly, in TOURMALINE-MM3, the evaluation of safety was not confounded by the contribution of AEs or overlapping toxicities from other agents in a combination regimen. Additionally, due to the direct comparison to placebo, the TOURMALINE-MM3 results provide an unbiased illustration of the safety of long-term single-agent ixazomib and demonstrate the tolerability and manageable toxicity profile of ixazomib maintenance therapy in patients who have undergone ASCT.

After receiving ASCT, responding patients are likely to be symptom-free, and transplant-related toxicities are generally expected to have resolved before maintenance treatment is initiated. Given the potential for prolonged therapy, a maintenance treatment with minimal cumulative toxicity, such as irreversible PN or nominal bone marrow function decline, that could impact later lines of therapies is desirable. In TOURMALINE-MM3, no evidence of cumulative toxicity or of long-term or late-onset toxicity was observed with ixazomib maintenance therapy. The rate of discontinuation due to AEs was similar between treatment arms (7% vs. 5%) compared with a rate of 29% previously reported in a meta-analysis of post-ASCT lenalidomide maintenance [8], although the mean lenalidomide treatment durations in the studies from which discontinuation rates were available were substantially longer than that for ixazomib in TOURMALINE-MM3. In addition, of the 594/1137 (52%) patients who had discontinued lenalidomide in the Myeloma XI study of lenalidomide maintenance versus observation in newly diagnosed multiple myeloma, the rate of discontinuation due to AEs with lenalidomide was 15% [18].

Gastrointestinal toxicity is a key consideration for patient management in the treatment of multiple myeloma, in particular with oral agents, and has been previously reported with ixazomib [15, 16]. Per protocol for TOURMALINE-MM3, standard antiemetics were recommended for emesis occurring once treatment was initiated, and prophylactic antiemetics were suggested for consideration at the physician’s discretion. Although the rates of gastrointestinal toxicities were higher with ixazomib versus placebo, the rates of grade ≥ 3 events were low in both groups and discontinuations due to gastrointestinal AEs were rare. The incidence rate of gastrointestinal AEs was higher in the first 3 months, and events were manageable with standard supportive care.

Hematologic toxicities, specifically thrombocytopenia events, were also manageable in this patient population. The transient cyclical thrombocytopenia reported with ixazomib is a known class effect of proteasome inhibitors; it is likely a result of transient inhibition of nuclear factor κB signaling via inhibition of the 26S proteasome, which is one of the required signaling cascades for platelet budding from megakaryocytes [19]. The mean platelet count remained generally constant over time with ixazomib in TOURMALINE-MM3, and very few patients had new-onset thrombocytopenia later in their treatment course. There were also 2 patients receiving placebo who reported grade ≥ 3 thrombocytopenia. In the ixazomib arm, the events of thrombocytopenia that did not spontaneously resolve were readily managed using dose interruptions/reductions and rarely required transfusions.

PN is an important side effect in the treatment of multiple myeloma [20]; it can be caused by the disease itself or by specific agents, in particular bortezomib and thalidomide [20–22]. In TOURMALINE-MM3, PN rates were similar in the ixazomib and placebo arms and events were typically of low grade. Interestingly, the cumulative incidence of all grade PN was similar in both arms for the first 5 months and then gradually became higher with ixazomib treatment compared with placebo. The incidence of prior PN is a known predisposing factor for development of PN and may be impacted by prior bortezomib or thalidomide treatment [20]. However, analyzing the incidence of PN by prior proteasome inhibitor therapy or prior thalidomide, or by presence of PN at study entry, demonstrated no consistent patterns across treatment arms. PN reported with ixazomib maintenance was effectively managed with concomitant pain medications, primarily pregabalin and gabapentin, and PN events reported with ixazomib were reversible in the majority of cases.

Infections are not unexpected in patients with multiple myeloma following ASCT [23]. Upper respiratory tract infections were among the most frequently reported AEs with ixazomib, but these events were largely manageable. Clinicians should be aware of the risk of pneumonia and the increased risk of herpes zoster reactivation in the absence of antiviral prophylaxis. However, the rate of herpes zoster was low in patients receiving prophylaxis—indeed, antiviral prophylaxis virtually eliminated the risk of herpes zoster reactivation and should always be administered with ixazomib unless there is a clinical contraindication.

The risk of venous thromboembolism is approximately 3 to 10% in patients with multiple myeloma [24]. Venous thromboembolism prophylaxis strategies are recommended for patients receiving specific agents, such as lenalidomide; however, to date, the use of proteasome inhibitors has not been associated with thromboembolism [24, 25]. As such, in TOURMALINE-MM3, thromboprophylaxis was not required per protocol but could have been administered per institutional guidelines, with 19% of patients in each arm receiving an antithrombotic agent. Ixazomib maintenance therapy was not associated with an increased risk of thromboembolism compared with placebo in this study.

In summary, this study has shown that single-agent ixazomib is well-tolerated and that long-term maintenance treatment is feasible post-ASCT. AEs associated with ixazomib maintenance can be managed in the context of routine post-ASCT supportive care due to the limited additional toxicity. Ixazomib is an efficacious and tolerable option for post-ASCT maintenance in patients with newly diagnosed MM.

## Data Availability

Takeda makes patient-level, de-identified datasets and associated documents available after applicable marketing approvals and commercial availability have been received and other criteria have been met as set forth in Takeda’s Data Sharing Policy (see https://www.takedaclinicaltrials.com for details). To obtain access, researchers must submit a legitimate academic research proposal for adjudication by an independent review panel, who will review the scientific merit of the research and the requestor’s qualifications and conflict of interest that can result in potential bias. Once approved, qualified researchers who sign a data-sharing agreement are provided access to these data in a secure research environment.
